# Sexual assault while too intoxicated to resist: a general population study of Norwegian teenage girls

**DOI:** 10.1186/1471-2458-14-406

**Published:** 2014-04-28

**Authors:** Hilde Pape

**Affiliations:** 1 Norwegian Institute for Alcohol and Drug Research (SIRUS), PB 565 Sentrum, 0105 Oslo, Norway

**Keywords:** Sexual assault victimization, Alcohol, Incapacitation, Impulsivity, Behavioral problems, Teenage girls, Prevention

## Abstract

**Background:**

Underage drinking is widespread, but studies on alcohol-related sexual victimization among teenage girls are almost non-existent. Research on individual correlates and risk factors of sexual victimization more generally is also meager. This study focuses on sexual assault while incapacitated due to drunkenness among 15–18 year-old girls and examines how age, drinking behavior, impulsivity and involvement in norm-violating activities are associated with such victimization experiences.

**Methods:**

Data stemmed from a school survey (response rate: 85%) in 16 Norwegian municipalities. Almost all analyses were restricted to girls who had been intoxicated in the past year (n = 2701). In addition to bivariate associations, adjusted odds ratios and relative risks of incapacitated sexual assault (ISA) were estimated. Further, population-attributable fractions were calculated to explore how the prevalence of ISA victimization was likely to be affected if effective preventive measures were targeted solely at high-risk groups.

**Results:**

The majority of the girls (71%) had been intoxicated in the past year, of which 7% had experienced ISA victimization in the same period. The proportion of victims decreased by age within the group that had been intoxicated, reflecting that the youngest girls were more likely to get severely drunk. Impulsivity and involvement in norm-violating behaviors were identified as potential risk factors, but the population-attributable fractions indicated that the groups with the highest risk of ISA victimization accounted for only a minority of all the cases of such victimization.

**Conclusion:**

Sexual assault against teenage girls who are too drunk to resist seems to be prevalent in Norway – notably among the youngest girls who engage in heavy episodic drinking. This study also suggests that one should reconsider the notion that no individual attributes are related to females’ sexual assault victimization. It also indicates that a high risk approach to prevention, targeting groups with a high level of impulsivity or behavioral problems, may have limited effect on the prevalence of ISA victimization. Thus, from a public health perspective, it may be advisable to give priority to universal preventive measures to curb young girls’ risk of being sexually assaulted in a state of alcohol-induced incapacitation.

## Background

Underage drinking is prevalent in Europe and North-America [1,2], but studies on alcohol-related sexual assault victimization among teenage girls are almost non-existent. In contrast, numerous studies of young adult women have addressed the issue and the bulk of this research has been conducted on samples of US female college students, e.g. [3-6]. Among other things, it has been shown that the risk of sexual victimization is higher on drinking days as compared to non-drinking days [7,8], and increases with the level of blood alcohol concentration [9]. In part, this probably reflects that alcohol may impair the ability to acknowledge potential danger and to effectively fend off unwanted sexual advances. Moreover, incapacitation may occur when large quantities are consumed, and in such a vulnerable state one may be taken advantage of sexually while unable to object or physically resist.

Studies of US college women indicate that incapacitated sexual assault (ISA) is the major type of sexual assault victimization in this student population [10]. For instance, a national college-based survey showed that 72% of the female students who reported being raped were incapacitated due to excessive drinking when it occurred [11]. The risk of ISA seems to be elevated among the youngest college women [11-13] but it has not been reported whether this may reflect age-related changes in lifestyle and drinking behavior.

The research literature seems to include only four studies of alcohol-related sexual assault victimization among teenage girls, and they were all conducted in the USA. One of them showed that sexual victimization was related to the frequency binge drinking, yet this association disappeared when excluding girls who reported ISA [14]. Another study assessed whether the victim and/or the perpetrator had used alcohol at the time of the assault, but no data on victim incapacitation due to excessive drinking were collected [15]. Such data were available in a national survey of 13–19 year-olds from the late 1970s, but the exact prevalence of ISA victimization was not reported [16]. However, it was low (0%-4%) and no further analyses were undertaken. Another study of a US national sample recently showed that two per cent of the girls aged 12–17 years had ever been sexually assaulted while under the influence of substances, and that a solid majority (68%) of the cases involved endorsements of being “too drunk to know what was happening” [17]. The proportion of girls who had ever been intoxicated was not given, neither was the prevalence of ISA victimization within that group.

Analyzing data from a large-scale survey of senior high school students in Norway, the present study adds to the meagre research literature on ISA victimization among teenage girls. It also expands the body of research on individual correlates of sexual victimization, focusing specifically on impulsivity and involvement in norm-violating behaviors. The issue of victim-related risk factors is sensitive and publications reporting that heavy drinking increases vulnerability typically emphasize that the victims are not responsible for the sexual assaults perpetrated against them, e.g. [5,10,13,18]. Indeed, the distinction between identifying potential risk factors and attributing blame is essential and should be kept in mind.

Because the proportion of heavy episodic drinkers sets a limit for the occurrence of ISA victimization, it can be inferred that the prevalence is very low among girls in their early teens and escalates thereafter. The association with age may be different within the group of girls who drink to intoxication. The youngest teenage girls are generally less cognitively mature and also less experienced with sex and dating. Hence, their ability to anticipate, recognize and respond to sexual assault risk is probably more limited than that of older teenage girls. They are also less likely to be experienced drinkers and to know their limits, and may to a greater extent drink far too much in potentially risky situations. Therefore, one may assume that the vulnerability to ISA is inversely related to age among teenage girls who engage in heavy episodic drinking. This assumption was tested in the present study.

Apart from investigations on the role of alcohol, research on individual correlates and vulnerability factors of sexual assault victimization is sparse. A review of the literature concluded that no specific personality attributes seem to be associated with such victimization [4], but this conclusion may be questioned due to the paucity of studies addressing the issue. Moreover, traits of potential importance have barely been examined in this context.

Numerous studies have shown that impulsivity and related personality characteristics (e.g. low self-control) are associated with hazardous drinking and involvement in other deviant and health-risk behaviors [19-23]. A link to various forms of criminal victimization has also been found [24-26], suggesting that a tendency to disregard the potentially harmful consequences of one’s actions may put individuals at risk. Moreover, a recent study of college women showed that low self-control greatly increased the odds of an affirmative answer to the question “Have you ever found out that a man had obtained sexual intercourse with you by getting you drunk or high?” [27]. The respondents had volunteered for the study and it was thus recommended to pursue the issue in studies based on more representative samples.

Focusing on impulsivity and involvement in behaviors that may be indicative of weak internal constraints (e.g. aggression), the present study met this request. Whether engagement in deviant behavior more generally is associated with ISA victimization was also examined. It has been proposed that adolescent girls’ vulnerability to sexual assault may be linked to such behaviors, among other things through an increased likelihood of socializing with potential perpetrators in contexts with no supervising adults present [28]. Moreover, a survey of 16–20 year-old girls showed that indicators of a risky lifestyle (e.g. drug use, involvement in fights and risky sexual behavior) correlated with sexual victimization [29]. An association with illegal substance use has also been found in studies focusing specifically on ISA victimization among college women [11,30].

More research on potential risk factors for sexual assault victimization is warranted, not least because the results may have implications for prevention. However, the prevention paradox [31,32] may apply in the current context, implying that the high-risk groups account for only a fraction of all cases of ISA victimization. Because the low-risk groups include far more individuals than the high-risk groups, their sheer size may make up for the lower risk of victimization at the individual level.

### Aims

Analyzing data from a large sample of senior high school students in Norway, the present study aimed at testing the assumption that ISA victimization is inversely related to age among teenage girls who drink to intoxication. How impulsivity and involvement in norm-violating behaviors were associated with such victimization experiences, and whether the prevention paradox made itself evident, were also examined.

## Methods

In the period 2004–2006, the Norwegian Institute for Alcohol and Drug Research conducted annual school surveys in 16 municipalities, covering both rural and urban areas of the country. The target samples comprised full cohorts of students and the major initial purpose was to evaluate a community prevention project aimed at reducing alcohol use and related harm. The evaluation study found no indications that such effects were achieved [33].

Data from the 2006 survey of the senior high school students were used in the present study. There were 31 such schools in the municipalities of which 28 took part in the study. The response rate at the participating schools was 85%. By mistake, eight schools received the junior high school version of the questionnaire – which did not assess ISA victimization. Our analyses were thus based on data from students at 20 senior high schools, and the study sample was restricted to girls aged 15–18 years. About 2% of these girls did not answer the question about ISA victimization and were therefore excluded. All girls in the remaining sample (N = 3784) were included when describing the prevalence of ISA, while all other analyses were confined to those who had been intoxicated in the past year. This group comprised 2701 girls whose mean age was 16.8 years (SD = 0.87). Their ethnic background showed little variation, i.e. 98% reported that their parents were born in Norway or in another Nordic country.

The study was conducted in accordance with the national guidelines for research ethics in the social sciences and approved by the Norwegian Social Science Data Services (NSD). NSD assesses research projects on behalf of the Norwegian Data Inspectorate and ensures that the data subjects’ rights are safeguarded. In Norway, approval from an ethics committee is only required for medical research, but studies that collect sensitive data and/or information about individuals that are stored electronically must be approved by NSD. Detailed information about the design, procedures and data collection strategy of the survey on which the present paper is based is reported elsewhere [34].

### Measures

An item about ISA victimization was included in a battery of questions about problems and negative experiences in relation to drinking during the past 12 months. The respondents were asked how many times they had been taken advantage of sexually without being able to resist because they were very drunk. A dichotomous variable (0 versus 1+ times) was used in the analyses.

Two indicators for heavy episodic drinking during the past 12 months were used; the frequency of intoxication and a severe drunkenness scale. The former was captured by asking the respondents how often they had “felt clearly intoxicated”. The answers were recorded on a 7-point scale and a semi-continuous variable was constructed by applying the following scale transformation: No times (coded 0), 1–4 times (2), 5–10 times (7.5), about once a month (12), 2–3 times a month (30) about once a week (52) and more than once a week (104). Moreover, the frequency of alcohol-induced blackout (“been somewhere without remembering how you got there”) and motor impairment (“got so drunk that you could not stand upright”) was assessed on this scale: Never (coded 0), once (1), 2–4 times (3), 5–10 times (7.5), 11–20 times (15.5), and >20 times (25). The drunkenness scale was constructed by adding up the two semi-continuous variables.

The questionnaire included six items from Plutchik and Van Praag’s [35] impulsivity scale. The original 15-item scale has been tested in both clinical and college student samples [35-37], and the selection of the six items was based on previous general population studies of adolescents [38,39]. The respondents considered statements such as “I act on the spur of the moment” and “It is hard to control my feelings”, and reported how often they experienced that this was the case. There were four response options; almost never (coded 1), sometimes (2), often (3) and very often (4). A summary measure was constructed by averaging each respondents’ scores for the six items (Cronbach’s Alpha = 0.61). A dichotomous variable was also used in some analyses. It was constructed by separating respondents with a high level of impulsivity (e.g. scores ≥ 3.0) from all others.

Involvement in norm-violating activities during the past 12 months was measured with dichotomous variables (yes/no) of violent behavior (“hit or kicked somebody”), vandalism (“deliberately broken or destroyed objects”), bullying (“ostracized or tormented fellow students”), minor theft (“stolen something worth < NOK 500” (≈90 USD)), truancy (“skipped school one whole day”) and illegal drug use. The six items were added up, thus yielding a measure on the diversity of engagement in norm-violating behavior. Previous research based on similar items showed that this kind of behavioral diversity is a better indicator for the seriousness of delinquent involvement among adolescents than are various sub-dimensions of deviant behavior [40].

### Statistical analyses

Differences between proportions were examined using χ^2^-tests while analyses of variance with F-tests were used to examine differences between group means. Zero-order correlations were assessed using Pearson’s r and univariate logistic regression, while multiple logistic regression analyses were applied to estimate associations that were adjusted for the respondents’ age and drinking behavior. Moreover, adjusted odds ratios of ISA victimization for respondents with a high level impulsivity and for groups with different levels of involvement in norm-violating behaviors at various levels of impulsivity and involvement in norm-violating behaviors were converted into relative risks (RRs) applying standard procedures [41]. In the next step, the RRs were used to calculate population-attributable fractions (PAFs). In the present context, these fractions express the expected percentage reduction of the total prevalence of ISA victimization if preventive measures targeting individuals at a given level of impulsivity or involvement in norm-violating behaviors were 100% effective. The PAFs were calculated using the standard formula for multiple levels of risk exposure [42], i.e.

(1) $$\text{PA}F_{i}=p_{i}\times \left(RR_{i}-1\right)/RR_{i},$$

where *p*_*i*_ is the percentage of all cases at level *i,* and *RR*_*i*_ is the relative risk at the same level*.* It should be noted that the reason for applying RRs rather than ORs is that the former is a more appropriate risk measure when calculating PAFs. More details about this statistical approach are provided by Norström and Pape [43] who recently used the same approach in an analogue context.

## Results

ISA victimization during the past 12 months was reported by 5.3% of all the girls and 7.4% of the girls who had been intoxicated in the same period. The prevalence in the whole sample did not vary significantly with age (Table 1). However, an inverse association between age and ISA victimization emerged when the analyses were restricted to girls who reported at least one intoxication episode in the past year.

**Table 1:** Prevalence of ISA victimization during the past 12 months by age in the full sample and among girls who had been intoxicated in the same period

**Age**	**Number of victims**	**All girls**		**Girls who had been intoxicated**	
		**N**	**% victims**	**N**	**% victims**
15 yrs	10	175	5.7	95	10.5
16 yrs	77	1370	5.6	856	9.0
17 yrs	73	1306	5.6	986	7.4
18 yrs	41	933	4.4^ns^	764	5.4*

*p < 0.05.

The proportion of girls who had been intoxicated was 54% among the 15-year-olds, 63% among the 16-year-olds, 76% among the 17-year-olds and 82% among the 18-year-olds (p < 0.001). All subsequent analyses were confined to these girls (n = 2701). The majority of them (62%) had experienced symptoms of severe drunkenness (i.e. alcohol-induced motor impairment and/or blackout) during the past 12 months and it may be noted that such symptoms were reported by almost all the ISA victims (91%).

Further analyses of girls who had been intoxicated showed that the frequency of intoxication was significantly associated with ISA victimization (r = 0.20, p < 0.001), as was the severe drunkenness scale (r = 0.31, p < 0.001). Moreover, while the frequency intoxication tended to increase with increasing age (r = 0.08, p < 0.001), the severe drunkenness scale was inversely related to age (r = −0.09, p < 0.001). Hence, when mid-teen girls reach the point of feeling the intoxicating effects of alcohol, they are apparently somewhat more likely than older teenage girls to continue drinking and to get very drunk – which in turn may put them at risk of ISA victimization.

Indeed, calculations showed that 15.2% (184/1216) of all the episodes of intoxication that were reported by the 15-year-olds involved motor impairment, as did 11.9% (1448/12184), 9.3% (1378/14858) and 5.3% (695/13217) of those reported by the 16-, 17- and 18-year-olds, respectively (p < 0.001). A similar pattern was observed for alcohol-induced blackout. Moreover, logistic regression analyses showed that the unadjusted statistical effect of age on ISA victimization (OR = 0.76, 95% Cl = 0.65-0.91) declined and was no longer significant when the severe drunkenness scale was added to the equation (Adj. OR = 0.86, 95% Cl = 0.71-1.02).

Table 2 shows that girls who reported ISA victimization had significantly higher mean scores for the impulsivity scale than other girls. They were also much more likely to report perpetration of violent behavior, vandalism, bullying and theft, as well as illegal drug use and truancy from school. Correspondingly, the mean number of norm-violating behaviors was significantly higher among the victims than among the non-victims. The statistical impact of all these variables remained significant when age and both indicators for heavy episodic drinking were taken into account.

**Table 2:** Impulsivity, involvement in norm-violating behaviors and ISA victimization

	**Unadjusted means (SD) and proportions**		**Associations with ISA victimization adjusted for age and drinking behavior**^**1**^		
	**Not victims**	**Victims**	**B**	**SE**	**Adj. OR (95% CI)**
Impulsivity	2.60 (0.48)	2.30*** (0.44)	0.88	0.17	2.40 (1.72 - 3.35)***
Violence	22.1	49.5***	0.69	0.17	2.00 (1.43 - 2.79)***
Vandalism	9.1	26.3***	0.62	0.20	1.86 (1.25 - 2.77)**
Bullying	13.1	30.0***	0.52	0.18	1.68 (1.18 - 2.41)**
Theft	11.1	25.3***	0.48	0.20	1.62 (1.10 - 2.39)*
Illegal drug use	11.7	33.5***	0.75	0.19	2.11 (1.46 - 3.05)***
Truancy	51.1	75.5***	0.77	0.18	2.16 (1.52 - 3.07)***
Number of norm-violations	1.17 (1.21)	2.35*** (1.68)	0.34	0.06	1.41 (1.26 - 1.58)***
Lowest N	2480	194	2674		

^1^I.e. the frequency of intoxication and the severe drunkenness scale. *** p < 0.001 **p < 0.01 *p < 0.05.

The relative risk of ISA victimization was substantially elevated among respondents with a high level of impulsivity and increased markedly by the extent of involvement in norm-violating activities (Table 3). Moreover, the population-attributable fractions suggest that the groups with the highest risk of ISA accounted for a minority of all the cases of victimization. More precisely, the results indicate that if all cases of ISA victimization in the group scoring high on the impulsivity scale (i.e. the top 6%) were prevented, the expected reduction in the total prevalence of ISA would be about 10%. As regards involvement in norm-violating behaviors, the estimates indicate that the reduction in the prevalence of ISA victimization would be about 18% if all cases in the most extreme group (i.e. the top 7%) were prevented. If the cases in the group with the second highest risk (e.g. those reporting involvement in 2–3 norm-violations) were eliminated as well, the total reduction in the prevalence of ISA would be approximately 40%. These two groups comprised about one third of the girls in the sample.

**Table 3:** Relative risks (RR) and population-attributable fractions (PAF) of ISA victimization by the level of impulsivity and the number of norm-violating behaviors, controlling for age and drinking behavior

	**N**	**(%)**	**RR**	**PAF**
**Level of impulsivity**				
Low	2547	(94.3)	1.00	–
High	154	(5.7)	2.79	9.6
Sum	2701	(100)	–	–
**Number of norm-violations**				
None	881	(32.9)	1.00	-
1	881	(32.9)	2.07	14.1
2-3	723	(27.0)	2.51	22.1
4+	196	(7.3)	3.63	18.3
Sum	2681	(100)	–	54.9

## Discussion

Five per cent of the 15–18 year-old girls in this community-based study of senior high school students in Norway reported that they had been taken advantage of sexually while too drunk to resist during the past year. The solid majority (71%) had been intoxicated in the same period, of which seven per cent reported ISA victimization. Previous research on alcohol-related sexual assault against teenage girls is meagre and these findings clearly indicate that there is a substantial mismatch between the prevalence of ISA victimization and the body of research addressing the issue.

One may assume that the occurrence of ISA victimization among adolescent girls differs markedly cross-nationally – not least because of variations in drinking behavior. For instance, the ESPAD 2011 survey on substance use among 15–16 year olds in 36 European countries showed that the proportion of girls who had been intoxicated in the past month ranged from 6% to 38% [2]. The prevalence was 15% among Norwegian girls, which was identical to average prevalence at the country level. The laws regulating adolescent drinking also show cross-national variation. In Norway, it is prohibited to sell alcohol to youth below the age of 18, while consumption of alcoholic beverages by younger teenagers is not an offence. In the USA, as in other countries where alcohol use by minors is illegal, the perceived barriers against reporting incidents of ISA victimization to the police may be almost insuperable for underage girls. Conversely, the perpetrators’ actual as well as perceived risk of being charged and convicted is likely to be negligible.

In contrast to previous studies on alcohol-related sexual assault among teenage girls [15,17], the prevalence of ISA victimization in the present study did not increase by age, i.e. analyses of the whole sample revealed no significant association between the two. In this respect, the restricted age range of the sample should be kept in mind. On the other hand, the assumption that ISA is inversely related to age among girls who drink to intoxication was clearly supported; the prevalence within this group ranged from 5% among the 18-year-olds to 11% among the 15-year-olds. Thus, a remarkably high proportion of the mid-teen girls who engaged in heavy episodic drinking had been sexually assaulted while too drunk to resist in the past year.

In their study of adolescent girls’ experiences of sexual assault while under the influence of alcohol or drugs, McCauley and co-workers [17] examined whether victims and non-victims differed with respect to substance use. However, they did not take into account that all the victims – by definition – had started drinking or using drugs. The present study eliminated this source of bias by restricting all analyses of victim characteristics to girls who had been intoxicated. Within this group, measures of drinking behavior correlated quite weakly with ISA victimization. The youngest girls reported fewer intoxication episodes than the oldest, but were somewhat more likely to get severely drunk.

Further analyses indicated that the inverse association between age and ISA victimization among girls who had been intoxicated reflected variations in drinking style, i.e. it was no longer statistically significant when adjusting for symptoms of severe drunkenness. This, in turn, may reflect that younger adolescent girls are likely to be inexperienced drinkers and hence, that less consumption may be required to produce intoxicating effects. The youngest girls may also be less aware of what it takes to reach the point of severe drunkenness.

A high level of impulsivity significantly increased the odds of reporting ISA, as did involvement in norm-violating activities. The analyses included both aggressive and non-aggressive behaviors, and the pattern of findings was highly consistent. Drinking to incapacitation may in itself be indicative of weak internal constraints and a tendency towards norm-violation, but all associations with ISA victimization persisted when indicators for heavy episodic drinking were taken into account. These results corroborate those reported by Franklin [27], who found that weak self-control was independently associated with sexual assault victimization in relation to substance use among college women. The observed associations with norm-violating behaviors are also consistent with some previous studies on correlates of alcohol-related sexual assault victimization [11,29,30]. These findings may have various explanations and the cross-sectional design of the present study implies that they should be interpreted with caution.

By its very nature, impulsivity involves behavioral decision-making with no or little forethought of potential negative outcomes. Further, a tendency to act impulsively and to violate behavioral norms may increase vulnerability to sexual victimization due to a lifestyle that brings about exposure to risky places, situations and persons. Attachment to deviant peers is often embedded in such a lifestyle – which in itself has been found to be associated with an increased risk of ISA and other forms of personal victimization [27,44,45]. Moreover, there is evidence that impulsivity and related personality attributes are highly heritable and that various forms of personal victimization also may have genetic underpinnings [46,47].

On the other hand, impulsive behavior and acting-out tendencies might also be a potential consequence of individuals’ victimization experiences. It is well established that sexual assault may deteriorate the victims’ mental health and produce post-traumatic stress reactions [17,48,49]. There is also evidence of a link between exposure to traumatizing events and subsequent involvement in delinquency, and some studies suggest that this association is particularly evident for girls [50]. Further, much research, including studies focusing specifically on ISA [30,51], indicates that sexual victimization strongly increases the risk of subsequent sexual victimization [52-54]. The associations in question thus may reflect multiple contributing factors and complex reciprocal influences.

The population-attributable fractions indicated that a high risk approach to prevention, targeting groups with a high level of impulsivity or behavioral problems, may have limited effect on the total prevalence of ISA victimization among teenage girls. Thus, from a public health perspective, the results suggest that priority should be given to universal approaches to prevention. However, the reported findings are tentative, and it is possible that analyses of other potential risk factors would have led to a somewhat different conclusion.

The present study highlights the significance of implementing effective preventive measures to delay young people’s onset of alcohol use and to curb adolescent heavy episodic drinking. It is evidently important to restrict the opportunities for consuming alcohol in unsupervised settings, and interventions targeting parents may be particularly useful in this respect – not least because private homes are commonly used by adolescents as the location for drinking and getting drunk [55-57]. Moreover, there is some evidence that parent-based measures to reduce the risk of alcohol-involved sexual victimization among teenage girls may work as intended [58]. Prevention efforts should also target adolescent boys, yet the effectiveness of rape education programs in reducing the incidence of ISA and other kinds of sexual assault perpetration remains uncertain [59,60].

### Methodological considerations and suggestions for future research

Schools from geographically diverse regions of Norway took part in the current study and the response rate was high (85%). However, students in the biggest cities did not participate, which in part may explain the ethnic homogeneity of the study sample. Nearly all teenagers in Norway proceed to senior high school after graduating from junior high school, but a previous Norwegian study showed that heavy drinking predicted drop-out from senior high school [61]. Therefore, the issue of sample representativeness is left with some uncertainty.

In contrast to much previous research, we assessed the past year rather than the lifetime prevalence of sexual assault victimization, reducing the risk of memory distortion and recall bias. However, because no data on previous experiences of sexual assault were collected, the important issue of re-victimization could not be addressed. Moreover, the respondents were asked whether they “had been taken advantage of sexually” while too drunk to resist, and behaviorally specific questions tend to yield higher endorsement rates [62]. The nature and severity of the reported cases also remain unknown. Therefore, it should be noted that the Norwegian equivalent of the term “been taken advantage of sexually “denotes incidents of a more serious kind and that the item appeared in the context of other questions on sexual intercourse in relation to drinking. At any rate, more specific measures on ISA victimization would have been preferable. The issue should also be further scrutinized in qualitative studies – to uncover the stories behind the figures.

The respondents’ self-perceived intoxication was applied as an indicator for heavy episodic drinking. As discussed by Pape and co-workers [63], there is evidence that the feeling of being intoxicated correlates strongly with less subjective measures on alcohol use, including estimates of the level of blood alcohol concentration. It may be particularly hard for adolescents to estimate their alcohol consumption in terms of standard units, and Lintonen and Rimpelä [64] thus argued that one should rather rely on self-perceived intoxication as a simple and valid indicator of adolescent heavy drinking.

Only six items were used to construct the impulsivity scale and the inter-item reliability was quite low. The association with ISA victimization would most probably have been stronger if the internal consistency of this measure was higher. Thus, future research on potential risk factors for sexual assault victimization should include more comprehensive measures on impulsivity or related individual attributes.

Limitations due to the cross-sectional research design also warrant attention. The temporal order of impulsivity/norm-violating behaviour and ISA victimization could not be determined and longitudinal studies are required to scrutinize potential causal explanations for the observed associations. Moreover, such studies should cover a broader range of potential vulnerability factors than those assessed in the present study, including sexual victimization experiences prior to the onset of drinking, additional indicators for lifestyle, delinquent peer affiliation and other characteristics of the social network. Another suggestion for future research is to gain more knowledge about the issue of gender, drinking and dating, and the grey zones between consented and unconsented sex among adolescents. Thus, the situations in which unconsented sex occurs in relation to drinking may be more complex and ambiguous than one is given the impression of in the survey-based research literature on sexual assault victimization [65], but few studies have addressed the issue.

## Conclusion

This study strongly indicates that there is a mismatch between the prevalence of ISA victimization among teenage girls and the body of research addressing the issue. The proportion of victims was especially high among the youngest girls who engaged in heavy episodic drinking, providing yet another argument in favor of implementing effective preventive measures to postpone young people’s onset of drinking. Impulsivity and involvement in norm-violating behaviors were identified as potential vulnerability factors, suggesting that one should reconsider the notion that no specific individual attributes are related to females’ sexual assault victimization. The findings also indicate that it may be advantageous to implement universal measures to reduce the prevalence of ISA victimization in the population of teenage girls.

## Competing interests

The author declares that she has no competing interests.

## Pre-publication history

The pre-publication history for this paper can be accessed here:

http://www.biomedcentral.com/1471-2458/14/406/prepub
